# Analysis of Changes of Intestinal Flora in Elderly Patients with Alzheimer's Disease and Liver Cancer and Its Correlation with Abnormal Gastrointestinal Motility

**DOI:** 10.1155/2021/7517379

**Published:** 2021-08-13

**Authors:** Weiwei Zhang, Xuelian Zhang, Yanyan Zhang, Huanyu Wu, Qiaoling Liu, Xinyu Zhou, Yunxia Meng

**Affiliations:** ^1^Department of Geriatrics, The First People's Hospital of Lianyungang, Lianyungang 222000, China; ^2^Department of Geriatrics, The First Affiliated Hospital of Kangda College of Nanjing Medical University, Lianyungang 222000, China

## Abstract

**Objective:**

To investigate the changes of intestinal flora in elderly patients with Alzheimer's disease and liver cancer and its correlation with abnormal gastrointestinal motility.

**Methods:**

From January 2018 to December 2020, 102 elderly patients with Alzheimer's disease and liver cancer were selected as the observation group. Eighty-nine healthy patients during the same period were selected as the control group. The two groups of intestinal flora (intestinal microbial diversity) were detected by real-time fluorescent quantitative PCR (RT-qPCR) and high-throughput sequencing. The two groups of serum motilin (MTL) and gastrin (GAS) levels were measured by the Hitachi automatic biochemical analyzer 7600. Pearson correlation analysis software was used to analyze the relationship between changes in the intestinal flora and gastrointestinal motility in elderly patients with Alzheimer's disease and liver cancer.

**Results:**

The contents of *Bifidobacteria* and *Lactobacilli* in the observation group were lower than those in the control group, and the contents of *Escherichia coli*, *Helicobacter pylori,* and *Streptococcus* were higher than those in the control group. The Chaol index and Shannon index in the observation group were higher than those in the control group. The gastrointestinal motility levels MTL and GAS of the observation group were higher than those of the control group. The results of Pearson correlation analysis showed that the Chaol index and Shannon index of elderly patients with Alzheimer's disease and liver cancer were positively correlated with MTL and GAS.

**Conclusion:**

Elderly patients with Alzheimer's disease and liver cancer often have changes in the intestinal flora, which are correlated with abnormal gastrointestinal motility. Strengthening the analysis of changes in patients' intestinal flora can enhance clinical medication knowledge and improve gastrointestinal motility in patients.

## 1. Introduction

Alzheimer's disease is a degenerative disease of the nervous system with relatively insidious onset and progressive development in the elderly. The clinical manifestations are memory impairment, aphasia, ignorance, impairment of visual and spatial skills, and executive dysfunction [1]. Current studies show that the onset of Alzheimer's disease in the elderly may be related to family history, head trauma, viral infection, etc., but its specific pathogenesis is still not clear. With the increasing aging of the population in China, elderly Alzheimer's disease incidence is on the rise [2, 3]. Liver cancer is a malignant tumor that occurs in the liver, and its incidence is mainly related to viral hepatitis, drinking, eating moldy food, and heredity. The clinical symptoms in the early stage of the disease lack typicality, and as the course of the disease prolongs, they usually manifest as pain, fever, and fatigue in the liver area. Previous studies have shown that intestinal flora belongs to the “second genome” of the human body and is also an important defense line of the body's immune system [4]. Its dynamic balance is the basis for maintaining the body's normal function, and it has essential physiological and pathological significance for the human body. In addition, the intestinal flora can also affect the body's protein, neurotoxicity, and neuronal absorption [5]. For patients with intestinal flora disorder or imbalance, it will not only aggravate the damage and loss of neurons in the brain but also cause gastrointestinal dysfunction, thereby aggravating the occurrence and development of the disease. However, there are few clinical studies on the relationship of intestinal microflora changes in elderly Alzheimer's disease patients with liver cancer and its correlation with gastrointestinal motility abnormalities [6, 7]. Therefore, this study selected elderly patients with Alzheimer's disease and liver cancer and healthy subjects to explore the changes of intestinal flora in elderly patients with Alzheimer's disease complicated with liver cancer and their correlation with gastrointestinal motility abnormalities.

## 2. Materials and Methods

### 2.1. Clinical Information

From January 2018 to December 2020 at the First People's Hospital of Lianyungang, Lianyungang, China, 102 elderly patients with Alzheimer's disease and liver cancer were selected as the observation group and 89 cases of healthy physical examination during the same period were included in the control group. There was no statistically significant difference in the general information of the two groups of patients, and they were comparable (Table 1). The study was approved by the ethics committee of the First People's Hospital of Lianyungang, Lianyungang, China, and the patient's informed consent was obtained.

### 2.2. Inclusion and Exclusion Criteria

Inclusion criteria: (1) patients who meet the diagnostic criteria for Alzheimer's disease according to the American Psychiatric Association “Diagnosis and Statistical Work Manual of Mental Disorders Fourth Edition Revised Edition” [8]; patients with liver cancer diagnosed by pathological tissue examination; (2) patients with age ≥60 years, with complete baseline and follow-up data; and (3) patients with complete intestinal flora and gastrointestinal motility examinations and who can tolerate them. Exclusion criteria: (1) patients with autoimmune diseases, language communication disorders, or other mental illnesses; (2) patients who have taken neurotrophic drugs, microecological agents, and cardiovascular diseases within the past month; and (3) patients with severe liver and kidney dysfunction, accompanied by autoimmune system diseases.

## 3. Method

### 3.1. Intestinal Flora Determination

#### 3.1.1. Determination of Intestinal Flora

The RT-qPCR method was used to detect the total load of the intestinal flora in the two groups, including two types of intestinal probiotics (such as *Bifidobacterium* and *Lactobacillus*) and six types of intestinal pathogenic bacteria (*Escherichia coli*, *Staphylococcus*, *Veillonella*, *Helicobacter pylori*, *Streptococcus*, and *Pseudomonas aeruginosa*) levels. 300 mg of feces (middle feces) after admission of both groups were collected, and we completed the extraction of fecal bacterial DNA according to the instructions of the fecal bacterial genomic DNA extraction kit (Tiangen Biochemical Technology Co., Ltd., Beijing) and the PCR reaction. The primer is designed by Shanghai Yingjun Biotechnology Co., Ltd. According to the test content, the measurement of relevant parameters was completed: 10 min at 30°C; 30 min at 42°C; 5 min at 99°C; and 5 min at 5°C. A total of 35 cycles are completed, the final 10 min is extended, and the temperature is 72°C.

A standard calibration and negative control are set up for each experiment. After the reaction is completed, the specificity of the PCR product is analyzed according to the melting curve, and the final product is placed for agarose gel electrophoresis with a concentration of 1.5%. The result is the bacterial copy coefficient using logarithm/g feces mentioned above [9, 10].

#### 3.1.2. Diversity Detection of Intestinal Flora

High-throughput sequencing was used to detect the intestinal flora (intestinal microbial diversity) of the two groups. Stool samples were obtained from the abovementioned samples, and the intestinal flora diversity test was completed by Shanghai Shenggong Biological Engineering Co., Ltd. The Chaol index and Shannon index were calculated to complete the relative abundance analysis of the flora [11].

### 3.2. Measurement and Correlation of Gastrointestinal Motility Level

#### 3.2.1. Collection of Blood Samples

3 mL of peripheral fasting blood was taken from the observation group in the morning of the next day after admission, and 3 mL of peripheral blood was taken from the control group took on the day of physical examination and centrifuged for 30 min at a speed of 2500 rpm. Samples were placed at low temperature for use after the serum separation.

#### 3.2.2. Detection Method

The Hitachi 7600 automatic biochemical analyzer was used to determine the levels of serum MTL and GAS in the two groups. The detection method was enzyme-linked immunosorbent assay (ELISA). All kits were purchased from the Beijing East Asia Institute of Immunology. All operations were completed in strict accordance with the instrument instructions [12, 13].

#### 3.2.3. Correlation Analysis

Pearson correlation analysis software was used to analyze the relationship between changes in the intestinal flora and gastrointestinal motility in elderly patients with Alzheimer's disease and liver cancer.

### 3.3. Statistical Analysis

SPSS24.0 software was used to process and analyze the data. The counting data were tested by *χ*^2^ and expressed by *n* (%). Measurement data such as intestinal flora content were in line with normal distribution and were performed by the *t* test and expressed by ($\bar{x}\pm s$). *P* < 0.05 was considered statistically significant.

## 4. Results

### 4.1. Comparison of Intestinal Flora between the Two Groups

The contents of *Staphylococcus*, *Pseudomonas aeruginosa,* and *Veillonella* in the two groups were not statistically significant (Table 2). The contents of *Bifidobacteria* and *Lactobacilli* in the observation group were lower than those in the control group, and the contents of *Escherichia coli*, *Helicobacter pylori*, and *Streptococcus* were higher than those in the control group (Table 2).

### 4.2. Comparison of the Diversity of Intestinal Flora between the Two Groups

The Chaol index and Shannon index of elderly patients in the observation group were higher than those in the control group (Table 3).

### 4.3. Comparison and Correlation Analysis of Gastrointestinal Motility between the Two Groups

The gastrointestinal motility levels MTL and GAS of the observation group were higher than those of the control group (Table 4). The results of Pearson correlation analysis showed that the Chaol index and Shannon index of elderly patients with Alzheimer's disease and liver cancer were positively correlated with MTL and GAS (Table 5).

## 5. Discussion

Alzheimer's disease occurs more frequently in older adults, and common pathogenic factors include gene mutation and unhealthy lifestyle, but the specific pathogenesis of the disease has not been clarified clinically [14]. Liver cancer is also a malignant tumor with a high incidence rate. It occurs in people over the age of 40, and it is also an important cause of death among Chinese residents. Studies have shown that the brain tissues of elderly patients with Alzheimer's disease and liver cancer have obvious pathological changes [15]. Patients are often accompanied by amyloid deposition and neurofibrillary tangles after the onset of disease, which can cause nerve cell atrophy and death, and a serious condition of patients can cause cognitive dysfunction [15]. At the same time, Alzheimer's disease and liver cancer can interact and exacerbate, affecting the prognosis of patients.

In recent years, more and more research results have confirmed that intestinal microecological disorders are more common in elderly patients with Alzheimer's disease. The imbalance of the intestinal flora after the onset of the patient can affect the neuro-immune-endocrine system through the flora-brain-gut axis, which can damage the structure and function of the central nervous system [16, 17]. For normal people, intestinal flora is in a state of dynamic balance, but the imbalance of intestinal flora caused by various reasons will lead to the occurrence of a variety of diseases, increasing the incidence of chronic enteritis, gastrointestinal ulcer, stroke, and other diseases [18]. In this study, the contents of *Bifidobacterium* and *Lactobacillus* in the observation group were lower than those in the control group. In comparison, the contents of *Escherichia coli*, *Helicobacter pylori,* and *Streptococcus* in the observation group were higher than those in the control group, indicating that the elderly Alzheimer's disease and liver cancer patients were often accompanied by intestinal flora disorders, resulting in a decrease in the number of beneficial bacteria and an increase in the number of harmful bacteria.

Previous studies have shown that when the intestinal microecology is unbalanced and beneficial bacteria are reduced, a large amount of endotoxin will be produced, which will damage multiple tissues and organs throughout the body and increase the risk of damage to the central nervous system [19]. In addition, the reduction of beneficial bacteria and the increase of harmful bacteria in the intestines can affect the neuroendocrine system of patients, leading to neurotransmitter disorders [20]. In this study, the Chaol index and Shannon index of observation group were higher than those in the control group. The gastrointestinal motility levels MTL and GAS of the observation group were higher than those of the control group. It shows that the intestinal tract of elderly patients with Alzheimer's disease and liver cancer has the characteristics of diversity and complexity, which can cause the reduction of beneficial bacteria, can lead to abnormal gastrointestinal function, and aggravate the occurrence and development of the disease.

It has been reported that the decrease of beneficial bacteria in the intestine and the increase of harmful bacteria can cause a microinflammatory state and cause systemic oxidative stress, which are independent risk factors for Alzheimer's disease [21]. It can be seen that there is a close connection between intestinal microecological disorder and the occurrence of Alzheimer's disease. Thus, the correlation analysis between the changes of the intestinal flora of patients and the level of gastrointestinal motility was conducted in this study. The results show that the Chaol index and Shannon index of elderly patients with Alzheimer's disease and liver cancer are positively correlated with MTL and GAS, indicating that there is a strong correlation between the changes of intestinal flora and gastrointestinal motility in elderly patients with Alzheimer's disease and liver cancer. Therefore, at a clinic for elderly patients with Alzheimer's disease and liver cancer, there is a need to strengthen the measurement of the changes in the intestinal flora of the patients, evaluate and predict the gastrointestinal motility level of the patients, and adjust the treatment plan according to the measurement results, so that specific treatment is provided. However, there are some limitations to this study. The ELISA kit has some defects (such as operation errors), which may lead to data deviation. The patients enrolled in this study all took drugs for a long time, which may cause some interference to the study results.

In summary, elderly patients with Alzheimer's disease and liver cancer are often accompanied by changes in intestinal flora, which are correlated with abnormal gastrointestinal motility. Strengthening the analysis of changes in patients' intestinal flora can provide potential basis for clinical medication use and improve patients' health.

## Figures and Tables

**Table 1:** Comparison of general data between the two groups.

Clinical data	Observation	Control	*χ*^2^/*t*	*P*
Gender			1.214	0.793
Male	61	51		
Female	41	38		
Age (years)	71.49 ± 6.38	71.64 ± 6.42	0.591	0.325
BMI (kg/m^2^)	23.52 ± 3.26	24.97 ± 3.31	1.025	0.782
Disease duration (years)	12.52 ± 3.21	—	—	—
Education			1.491	0.678
Junior high school and below	41	36		
High school	54	47		
High school or above	7	6		
Electroencephalogram (EEG) examination				
Mild diffuse abnormalities	43	—	—	—
Moderate abnormalities	40	—	—	—
Severe abnormalities	19	—	—	—

**Table 2:** Comparison of intestinal flora between the two groups (logarithm/g feces, $\bar{x}\pm s$).

Group	Observation (*n* = 102)	Control (*n* = 89)	*t*	*P*
*Staphylococcus*	4.39 ± 0.56	4.41 ± 0.58	1.394	0.785
*Escherichia coli*	8.63 ± 0.83	4.12 ± 0.43	7.451	≤0.01
*Helicobacter pylori*	8.81 ± 0.88	5.29 ± 0.52	9.229	≤0.01
*Streptococcus*	7.40 ± 0.79	3.25 ± 0.45	6.672	≤0.01
*Veillonella*	4.21 ± 0.56	4.23 ± 0.58	2.142	0.561
*Pseudomonas aeruginosa*	4.06 ± 0.51	4.08 ± 0.53	0.638	0.829
*Bifidobacteria*	2.59 ± 0.41	6.63 ± 0.64	5.982	≤0.01
*Lactobacilli*	1.02 ± 0.25	5.49 ± 0.42	9.142	≤0.01

**Table 3:** Comparison of intestinal flora diversity between the two groups ($\bar{x}\pm s$).

Group	Cases	Chaol index	Shannon index
Observation	102	543.59 ± 35.61	5.70 ± 0.53
Control	89	287.43 ± 26.98	3.71 ± 0.41
*t*	—	7.813	6.092
*P*	—	≤0.01	≤0.01

**Table 4:** Comparison of gastrointestinal motility levels between the two groups ($\bar{x}\pm s$).

Group	Cases	MTL (ng/L)	GAS (pg/mL)
Observation	102	83.23 ± 4.51	96.49 ± 6.63
Control	89	51.16 ± 3.20	70.32 ± 4.74
*t*	—	8.142	5.091
*P*	—	≤0.01	≤0.01

**Table 5:** Correlation analysis of intestinal flora and gastrointestinal motility in elderly patients with Alzheimer's disease and liver cancer (*r*, *P*).

Correlation	MTL	GAS
Chaol index	0.774 (≤0.01)	0.815 (≤0.01)
Shannon index	0.698 (≤0.01)	0.791 (≤0.01)

## Data Availability

All data generated or analyzed during this study are included in the published article. The datasets used and/or analyzed during the present study are available from the corresponding author on reasonable request.
